# Salidroside promoted osteogenic differentiation of adipose-derived stromal cells through Wnt/β-catenin signaling pathway

**DOI:** 10.1186/s13018-021-02598-w

**Published:** 2021-07-16

**Authors:** Xiao-hua Li, Fu-ling Chen, Hong-lin Shen

**Affiliations:** 1 Department of Pharmacy, People’s Hospital of Changshou, Chongqing, China; 2 Department of Orthopaedic, People’s Hospital of Hechuan, Chongqing, China; 3 Department of Joint Trauma Surgery, People’s Hospital of Changshou, No. 16, Beiguan, Fengcheng Street, Changshou, Chongqing, China

**Keywords:** Salidroside, Adipose-derived stromal cells, Wnt/β-catenin signaling pathway, Osteogenic differentiation

## Abstract

**Background:**

Bone disease causes short-term or long-term physical pain and disability. It is necessary to explore new drug for bone-related disease. This study aimed to explore the role and mechanism of Salidroside in promoting osteogenic differentiation of adipose-derived stromal cells (ADSCs).

**Methods:**

ADSCs were isolated and treated with different dose of Salidroside. Cell count kit-8 (CCK-8) assay was performed to assess the cell viability of ADSCs. Then, ALP and ARS staining were conducted to assess the early and late osteogenic capacity of ADSCs, respectively.

Then, differentially expressed genes were obtained by R software. Then, Gene Ontology (GO) and Kyoto Encyclopedia of Genes and Genomes (KEGG) pathway analysis of the differentially expressed genes were further analyzed. The expression of OCN, COL1A1, RUNX2, WNT3A, and β-catenin were measured by real-time PCR and Western blot analysis. Last, β-catenin was silenced by small interfering RNA.

**Results:**

Salidroside significantly increased the ADSCs viability at a dose-response manner. Moreover, Salidroside enhanced osteogenic capacity of ADSCs, which are identified by enhanced ALP activity and calcium deposition. A total of 543 differentially expressed genes were identified between normal and Salidroside-treated ADSCs. Among these differentially expressed genes, 345 genes were upregulated and 198 genes were downregulated. Differentially expressed genes enriched in the Wnt/β-catenin signaling pathway. Western blot assay indicated that Salidroside enhanced the WNT3A and β-catenin expression. Silencing β-catenin partially reversed the promotion effects of Salidroside. PCR and Western blot results further confirmed these results.

**Conclusion:**

Salidroside promoted osteogenic differentiation of ADSCs through Wnt/β-catenin signaling pathway.

**Supplementary Information:**

The online version contains supplementary material available at 10.1186/s13018-021-02598-w.

## Background

Bone disease causes short-term or long-term physical pain and disability. Osteoblast differentiation is a highly regulated process controlled by a complex signaling pathway [1, 2]. Imbalance between bone formation by osteoblasts and bone resorption by osteoclasts contributes to osteoporosis [3, 4].

At present, drugs for the treatment of osteoporosis can be aimed at promoting osteoblastic bone formation or inhibiting osteoclasts [5]. Among them, parathyroid hormone is the only drug approved by the US Food and Drug Administration to promote bone formation. However, parathyroid hormone can cause osteosarcoma, which limits its large-scale clinical application [6]. Therefore, people’s attention has shifted to natural compounds.

The rhizome and roots of *Rhodiola rosea* L. have long been used in traditional medicine of China. Salidroside (SR) is a main component of *Rhodiola rosea* L. and exhibits a variety of pharmacologic properties, including anti-inflammatory, anti-fatigue and anti-oxidant properties, anti-apoptosis, and hypoglycemic effects. Mao et al. [7] found that Salidroside contributes to anti-aging effect in D-galactose induced aging model. Moreover, Salidroside protects cardiomyocyte against hypoxia-induced death through HIF-1α and VEGF-mediated pathway [8]. Zhang et al. [9] revealed that salidroside has protective effects against Abeta (25-35)-induced oxidative stress, which might be a potential therapeutic agent for treating or preventing neurodegenerative diseases. Studies have shown that a variety of natural compounds can affect the differentiation of osteoblasts [10]. However, whether SR could promote bone formation is not yet known.

In the treatment of many different bone diseases, mesenchymal stem cells were widely used in cell therapy [11]. Mesenchymal stem cells have the potential to differentiate into many different cell types such as osteoblasts, chondrocytes, muscle cells, and adipocytes [12–14]. Stem cells can be separated from bone marrow, synovium, periosteum, and fat tissue [14–16]. Among them, the source of adipose stem cells is abundant and easy to obtain, with little trauma [17]. Moreover, adipose stem cells proliferate faster than bone marrow stem cells. Therefore, adipose stem cells have more advantages than bone marrow stem cells in the cell therapy of bone diseases. Studies have shown that the adipose-derived stromal cells (ADSCs) have active biological properties, and its osteogenic differentiation are critical to the repair of bone loss and bone defect diseases [18]. Adipose-derived mesenchymal stem cells are easy to differentiate into adipocytes rather than osteoblast.

It was also reported that Wnt/β-catenin signaling is anabolic for bone formation [19]. The Wnt signaling pathway influences bone formation during development and bone remodeling during tissue renewal [20, 21]. Wnt inhibitors can promote preadipocyte differentiation by inhibiting Wnt/β-catenin signaling [22]. Therefore, the Wnt/β-catenin signaling pathway serves a notable role in osteogenic differentiation.

In this study, we performed RNA sequencing between normal and Salidroside-treated ADSCs. Moreover, we performed a series of experiments to verify that Salidroside promoted osteogenic differentiation of ADSCs through Wnt/β-catenin signaling pathway.

## Material and methods

### Isolation and culture of human adipose stem cells

Abdominal subcutaneous adipose tissue was obtained from human adipose tissue through simple liposuction. Then, 200–300 mL sterile PBS was added to every 0.5 g adipose tissue to prevent dehydration. Adipose tissues were cut into small pieces about less than 1 mm^3^ in size. Sterile saline (37 °C) was added to the homogenized adipose tissue in a ratio of 3:1 (saline to adipose tissue), followed by the addition of stock collagenase solution to a final concentration of 0.5 units/ml. This suspension was placed in a 37 °C shaker at 200 rpm for 60 min. Samples were then passed through a 100-μm filter into a 50-ml conical tube. After washing three times with sterile PBS, cells were then resuspend in high-glucose DMEM containing 20% FBS.

### Osteogenic differentiation of ADSCs

The third passage (P3) cells were used for all subsequent experiments. When 80% of the ADSCs were confluent, cells were seeded in 6-well plates and decomposed overnight. Then, ADSCs were changed to induction medium (10% high-glucose DMEM containing 10 mM β-glycerol phosphate, 10 nM dexamethasone, and 60 mM ascorbic acid). ADSCs were treated with osteogenic induction medium for 3 weeks and the medium was changed every 3 days.

### ALP staining and ALP activity

The ALP staining was performed using the ALP staining kit according to the instructions. In brief, ADSCs were fixed by 4% paraformaldehyde and then washed with PBS for three times. Staining was performed using BCIP/NBT chromogenic substrate (Vectastain ABC-AmP Kit, Vector Laboratories). An optical microscope (Olympus, Japan) was implemented for the staining result observation and photographing. ALP activity was measured according to the kit instructions. In brief, ADSCs were washed twice with TB buffer and then added 100 μL lysis solution. ADSCs were then separated for 20 min at 12,000 rpm at 4 °C. Then, 45 μL supernatant was added to 100 μL p-nitrophosphatate. After incubating for 30 min, the absorbance at 450 nm was measured with Microplate Reader (BioTek Instruments).

### Alizarin Red Staining

The content of calcium was measured by Alizarin Red Staining. In brief, 21 days after induction, ADSCs were fixed by 4% paraformaldehyde for 5 min and then washed by PBS three times. Then, ADSCs were incubated with 0.2% Alizarin Red solution for 30 min. Finally, optical microscope (Olympus, Japan) was implemented for the staining result observation and photographing.

### RNA sequencing

ADSCs underwent osteogenic differentiation and then divided into two groups: control and Salidroside-treated groups. ADSCs were treated with Trizol reagent to extract total RNA. Solexa pipeline v1.8 (Off-Line base Caller software, v1.8 Illumina, Foster City, CA, USA) was used to perform image analysis and substrate call. FastQC Software (Shanghai Kangcheng Biotechnology Co., Ltd.) was used to assess the quality and integrity of the RNA samples. HISAT2 software was used to align reads to the reference genome sequence (hg38). Transcripts from all samples were merged using StringTie to create a new reference file, which was then used to estimate transcript abundance in each sample. Differential expression analysis of the genes was performed using the DESeq package in R software (version 3.5.1). The heatmap of differentially expressed genes was drawn using the Complex Heatmap package in R. Gene ontology of the differentially expressed genes mainly including biological process, cellular component, and molecular function. Gene Ontology (GO) was performed using the clusterProfiler package in R software. The Kyoto Encyclopedia of Genes and Genomes (KEGG) pathway enrichment analysis of differentially expressed genes was performed using R software (version 3.5.1).

### Construction and amplification of recombinant adenoviruses expressing siβ-catenin

For constructing adenoviruses expressing siβ-catenin, siRNAs targeting the coding region of mouse β-catenin were assembled to an adenoviral shuttle vector. The siRNA target sites against human β-catenin-coding region were cloned into the pSES adenoviral shuttle vector to create recombinant adenoviruses. Polybrene (4 μg/mL) was used to increase adenovirus infection efficiency. The effects of the transfection on β-catenin reduction were assayed by Western blot analysis.

### Real-time polymerase chain reaction

Total RNA was extracted from ADSCs using the Trizol reagent in accordance with the manufacturer’s protocol. The first strand of cDNA was synthesized using the PrimeScript Reverse Transcriptase (RT) (TaKaRa Code: D2680S) and stored at − 20 °C for later use. The 7500 fast RT-PCR (ABI) was used for real-time quantitative PCR (qPCR). The expression of mRNA was calculated normalized to GAPDH with 2^−△△Ct^ method. The primers used were as follows: OCN, 5′-TCTTAGAACAAATTCTGCCCTTT-3′ (forward) and 5′-TGCTTTGGTCTTGAAATCACA-3′ (reverse); COL1A, 5′-CCTCCTCAGCTCACCTTCTC-3′ (forward) and 5′-GTTGGGAGCCCAAATAGAAA-3′ (reverse); RUNX2, 5′-AAGGTGTACGGCAAGGCTTC-3′(forward); 5′-CGTCAGAGCGAGTGAACCTC-3′ (reverse); GAPDH, 5′-CAATGTGGCCGAGGACTTTG-3′ (forward) and 5′-CATTCTCCTTAGAGAGAAGTGG − 3′ (reverse).

### Western blot

After washing human adipose-derived mesenchymal stem cells three times with PBS, total protein was extracted using RIPA protein lysis buffer (Beyotime, Shanghai, China) with freshly added 1% protease inhibitor cocktail and 1 mM phenylmethylsulfonyl fluoride (PMSF). Equal amounts of protein were resolved on a SDS-PAGE gel. After SDS-PAGE, the proteins were transferred to a PVDF membrane by electro-blotting for 1 h at 100 v. Membranes were blocked with 4% (w/v) non-fat milk powder in TBST (0.1% Tween-20 in Tris-buffered saline) at room temperature for 2 h. Then, the membranes were incubated with primary antibodies on a shaker, overnight at 4 °C. After that, the membrane was incubated with HRP-conjugated second antibody at room temperature for 2 h. The determination of the grayscale value was processed using ImageJ software.

### Statistical analysis

Statistical analysis was performed using the SPSS 20.0 statistical package (IBM Corp, Armonk, NY, USA). A statistically significant difference between groups was indicated at p < 0.05. Data are expressed as the mean ± standard deviation (SD). Differences were analyzed using one-way analysis of variance (ANOVA) followed by the Tukey’s post hoc test for multiple comparisons. Graphs were made using GraphPad Prism 7 (GraphPad Software).

## Results

### Salidroside enhanced cell viability of ADSCs

**Fig. 1:**
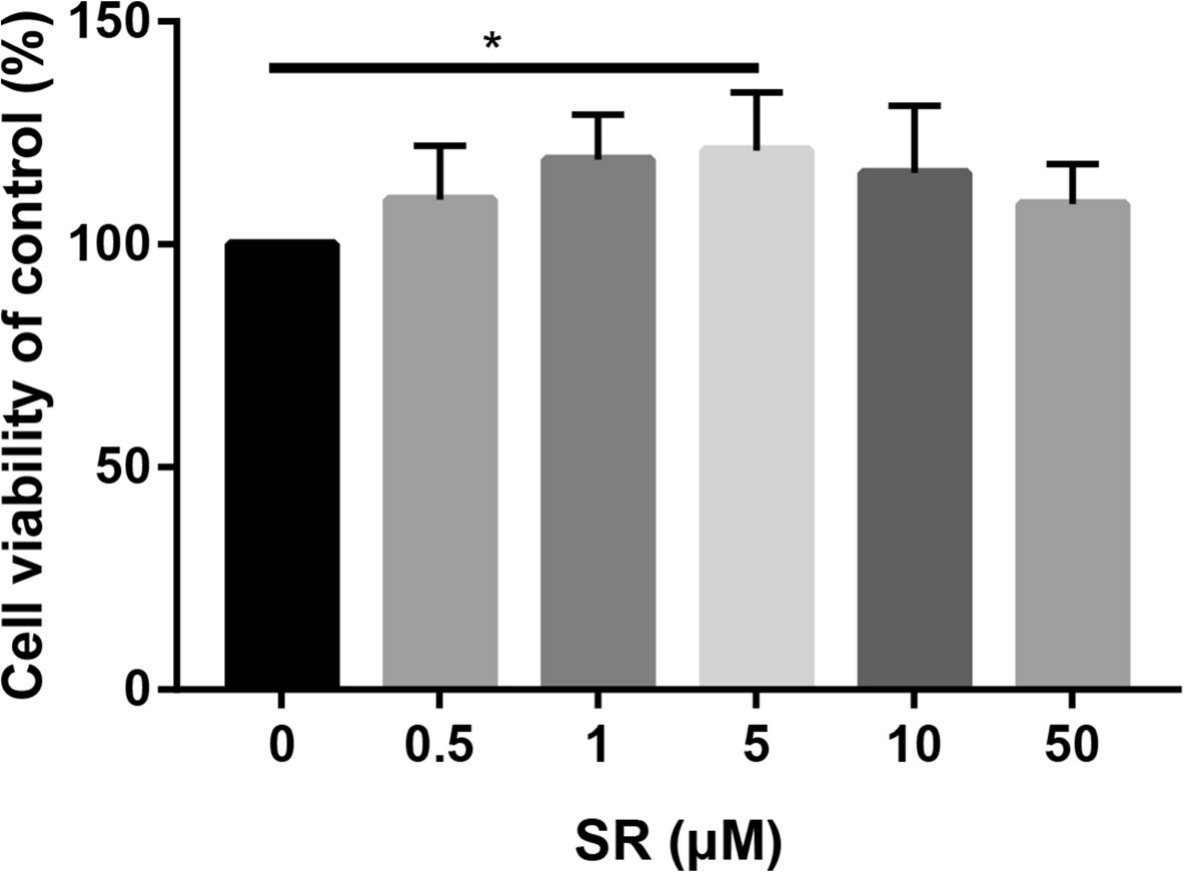
Cell viability of ADSCs in control and different dose of Salidroside was measured by CCK-8 assay

2D structure of Salidroside can be seen in Supplement S1. Salidroside As shown in Fig. 1, Salidroside slightly stimulated the proliferation of ADSCs within a 0.5–50-μM dose range, with 5 μM Salidroside showing maximal effect. However, the cell viability decreased slightly when the Salidroside concentration exceeded 5 μM.

### Salidroside enhanced osteogenic differentiation of ADSCs

**Fig. 2:**
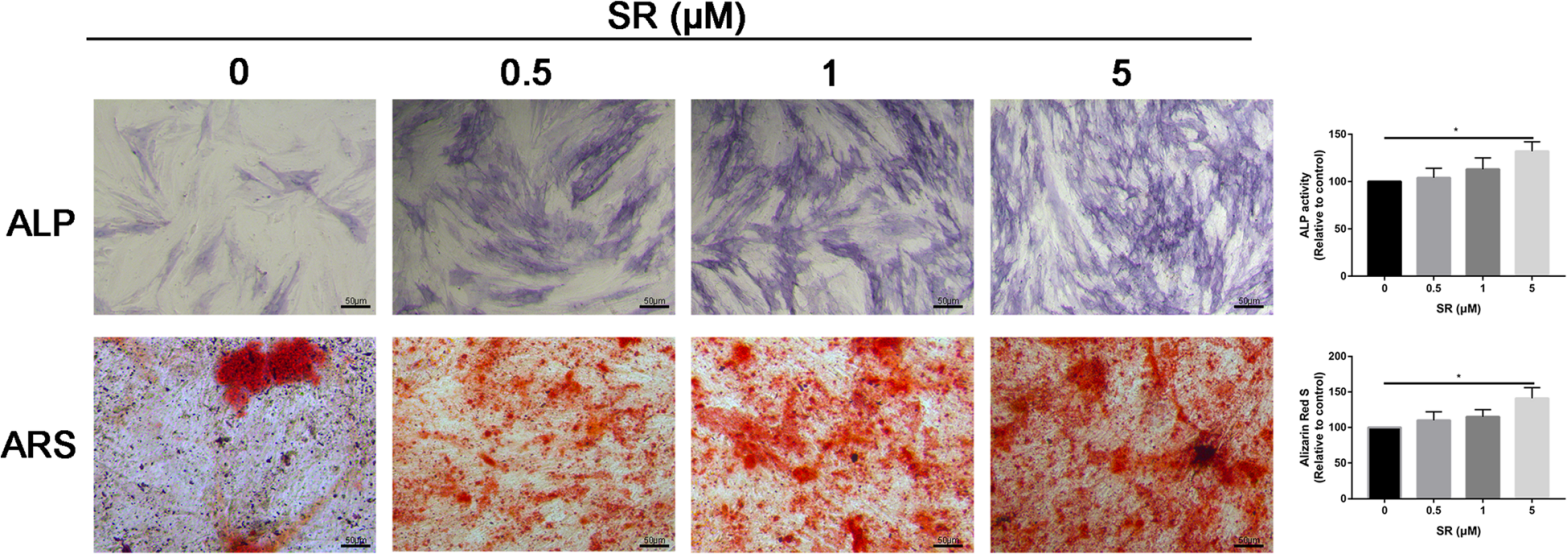
Salidroside promoted osteogenic differentiation of ADSCs. ALP and ARS staining for ADSCs in control and different dose of Salidroside-treated groups

**Fig. 3:**
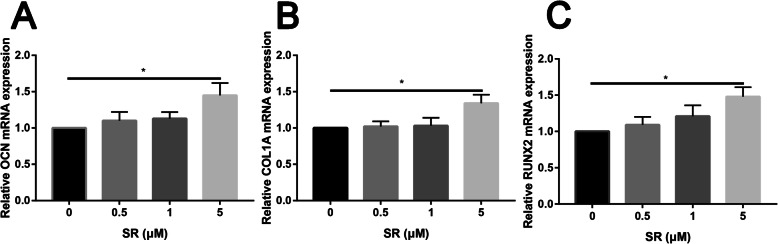
Real-time PCR analysis of OCN (**A**), COL1A1 (**B**), and RUNX2 (**C**) results of ADSCs in control and different dose of Salidroside-treated groups

In order to assess the role of Salidroside in promoting osteogenic differentiation of ADSCs, ALP, and ARS were performed to assess the early and late osteogenic capacity, respectively. As shown in Fig. 2, results indicated that Salidroside showed a dose-dependent osteogenic promotion effect after Salidroside treatment, including early and late osteogenic differentiation effects. Simultaneously, PCR analysis showed that the expression of the osteoblastic differentiation markers OCN (Fig. 3A), COL1A1 (Fig. 3B), and RUNX2 (Fig. 3C) were dose-dependently increased after Salidroside treatment; the difference was statistically significant (P < 0.05).

**Fig. 4:**
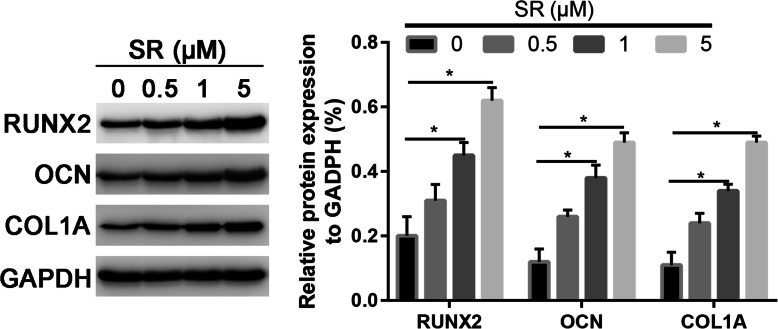
Western blot assay to assess the osteogenic markers in control and different dose of Salidroside-treated groups

Western blot analysis was in agreement with the quantitative real-time PCR (qRT-PCR) results, showing that the protein expression of OCN, COL1A1, and RUNX2 was upregulated in the Salidroside group (P < 0.05, Fig. 4).

### Bioinformatic analysis of Salidroside induced ADSCs

In order to identify the mechanism of Salidroside for promoting osteogenic differentiation of ADSCs. RNA sequencing and bioinformatic analysis were performed to reveal the mechanism of Salidroside in promoting osteogenic differentiation of ADSCs.

**Fig. 5:**
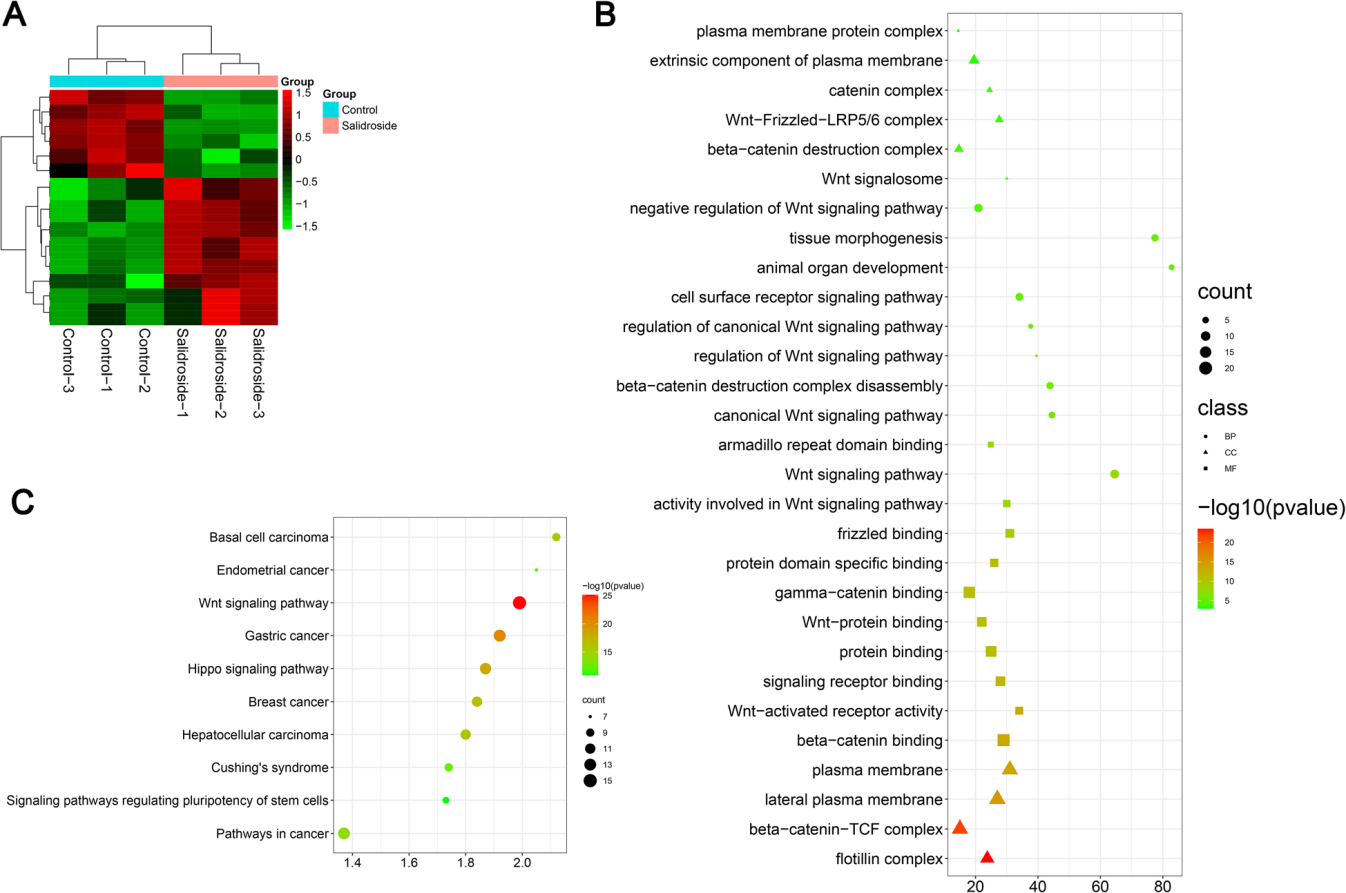
Identify the differentially expressed genes in control and Salidroside-treated ADSCs. **A** Heatmap of the top fourteen differentially expressed genes between control and Salidroside-treated ADSCs. **B** Gene Ontology of the differentially expressed genes. **C** KEGG pathway of the differentially expressed genes

A total of 543 differentially expressed genes were identified between normal and Salidroside-treated ADSCs. Among these differentially expressed genes, 345 genes were upregulated and 198 genes were downregulated. Top fourteen differentially expressed genes can be seen in Fig. 5A.

Gene ontology was divided into three categories: biological process, cellular component, and molecular function. Biological process of the differentially expressed genes mainly including Wnt signaling pathway, canonical Wnt signaling pathway, beta-catenin destruction complex disassembly, regulation of Wnt signaling pathway, regulation of canonical Wnt signaling pathway, cell surface receptor signaling pathway, animal organ development, tissue morphogenesis, and negative regulation of Wnt signaling pathway (Fig. 5B).

Cellular component of the differentially expressed genes mainly including Wnt signalosome, beta-catenin destruction complex, Wnt-Frizzled-LRP5/6 complex, catenin complex, extrinsic component of plasma membrane, plasma membrane protein complex, flotillin complex, beta-catenin-TCF complex, lateral plasma membrane, and plasma membrane (Fig. 5B).

Molecular function of the differentially expressed genes mainly including beta-catenin binding, Wnt-activated receptor activity, Wnt-protein binding, protein binding, gamma-catenin binding, protein domain specific binding, signaling receptor binding, frizzled binding coreceptor activity involved in Wnt signaling pathway, and armadillo repeat domain binding (Fig. 5B).

KEGG pathway mainly including Wnt signaling pathway, gastric cancer, hippo signaling pathway, breast cancer, hepatocellular carcinoma, basal cell carcinoma, pathways in cancer, Cushing’s syndrome, endometrial cancer, and signaling pathways regulating pluripotency of stem cells (Fig. 5C).

### Salidroside significantly activated Wnt/β-catenin signaling pathway

**Fig. 6:**
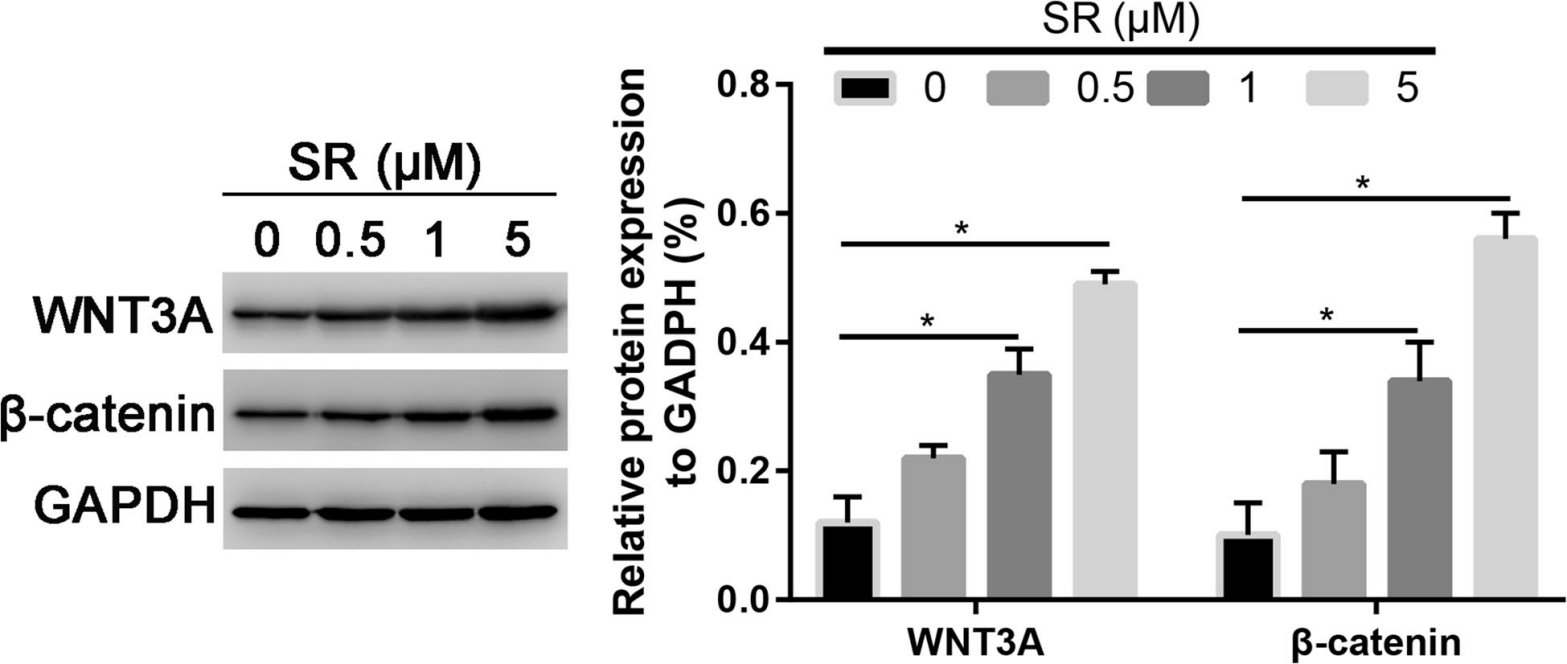
Salidroside enhanced osteogenic differentiation of ADSCs through Wnt/β-catenin signaling pathway. Western blot assay that assessed the WNT3A and β-catenin in control and Salidroside-treated ADSCs

In order to assess the mechanism of Salidroside in promoting osteogenic differentiation of ADSCs. We selected Wnt/β-catenin signaling pathway for further analysis. We found that Salidroside significantly increased the Wnt3A and β-catenin expression than control group (Fig. 6, P < 0.05).

### Silencing β-catenin partially reversed the osteogenic promotion effects of Salidroside

In order to further confirm the Wnt/β-catenin signaling pathway involved into the Salidroside promoted osteogenic differentiation of ADSCs.

**Fig. 7:**
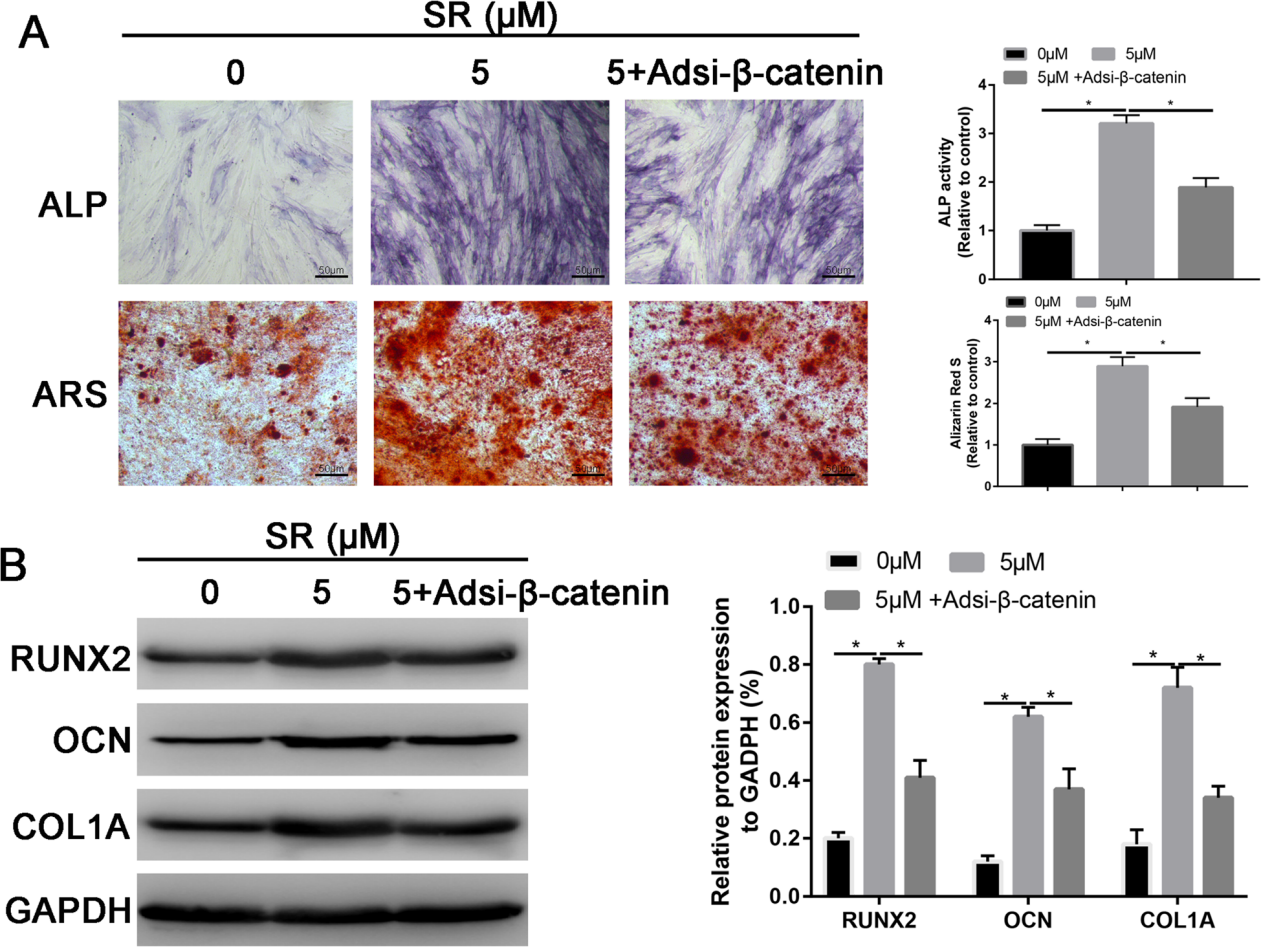
Silencing β-catenin partially reversed the promotion effects of Salidroside. **A** ALP and ARS staining in control, Salidroside, and Salidroside + si-β-catenin groups. **B** Western blot assay of the osteogenic markers in control, Salidroside, and Salidroside + si-β-catenin groups

ALP and ARS were then performed to assess the role of Salidroside for promoting osteogenic differentiation of ADSCs. We found that silencing β-catenin partially reversed the ALP activity and calcium deposition that induced by Salidroside (Fig. 7A).

Western blot analysis showing that the protein expression of OCN, COL1A1, and RUNX2 was partially reversed by silencing of β-catenin (P < 0.05, Fig. 7B).

## Discussion

In this study, we identified that Salidroside promoted osteogenic differentiation of ADSCs through Wnt/β-catenin signaling pathway. We firstly performed cell count kit-8 (CCK-8) assay to identify the optimal dose range of Salidroside. Then, we performed ALP and ARS staining assays to identify the osteogenic promotion effects of Salidroside. RNA sequencing was then performed to identify the differentially expressed genes between normal and Salidroside-treated ADSCs. Differentially expressed genes mainly enriched in the Wnt/β-catenin signaling pathway. We finally constructed small interfering RNA of β-catenin to verify the mechanism of Salidroside.

Strengths of this study were as follows: (1) we firstly performed RNA sequencing to assess the mechanism of Salidroside in promoting osteogenic differentiation of ADSCs, and (2) results revealed that Salidroside promoted osteogenic differentiation of ADSCs through Wnt/β-catenin signaling pathway.

The process of bone formation includes the initial proliferation of osteoblasts and the development and maturation of the extracellular matrix and the final mineralization. The osteogenic differentiation starts from adhesion and proliferation of several cell types, such as mesenchymal stem cells, pre-osteoblasts, and osteoblasts. Firstly, we performed CCK-8 assay to identify the optimal dose of Salidroside. We selected 5 μM Salidroside for further study. Then, ALP and ARS staining were performed to assess the osteogenic capacity of Salidroside. We found that Salidroside significantly increased osteogenic capacity of ADSCs. This study was in accordance with a previous study, which found that Salidroside protect against bone loss induced by H_2_O_2_ [23]. Bai et al. [24] revealed that Salidroside obviously promotes the proliferation of mesenchymal stem cells. These results suggested that Salidroside may be as alternative targets for osteoporosis therapies.

One of the commonly applied methodologies is deep RNA sequencing of proliferating, which indicated changes in the mRNA expression profiles in treatment and control groups. A total of 543 differentially expressed genes were identified between normal and Salidroside-treated ADSCs. Most of the differentially expressed genes mainly enriched in Wnt/β-catenin signaling pathway. Thus, we assessed the Wnt/3A and β-catenin expression in control and Salidroside-treated ADSCs. We found that Salidroside significantly increased Wnt/3A and β-catenin expression. Wnt/β-catenin was involved into multiple cells differentiation including neural differentiation [25], preadipocyte differentiation [26], and enterocyte differentiation [27].

In order to further identify the Wnt/β-catenin involved into the process of osteogenic differentiation of ADSCs. We constructed siRNA of β-catenin to inhibit the Wnt/β-catenin signaling pathway. We found that silencing β-catenin partially blocked the promotion effects of Salidroside on the osteogenic differentiation of ADSCs. Therefore, we concluded that Salidroside promoted osteogenic differentiation of ADSCs through Wnt/β-catenin signaling pathway.

This study has several shortcomings that are worth mentioning. First, the present study did not perform in vivo experiments. Second, receptor of Salidroside did not identify in this study. Third, the optimal dose regimen of Salidroside has not been established.

## Conclusion

In conclusion, our data indicate that Salidroside promoted osteogenic differentiation of ADSCs via the canonical Wnt/β-catenin signaling pathway. The findings in this study may provide insights into understanding the mechanism of Salidroside for osteogenesis. Salidroside may be a novel therapy for bone-related diseases.

## Supplementary Information

**Additional file 1: Supplement S1**: 2 D structure of Salidroside.

## Data Availability

We state that the data will not be shared since all the raw data are present in the figures included in the article.

## Abbreviations

ADSCs: Adipose-derived stromal cells
CCK-8: Cell count kit-8
GO: Gene ontology
KEGG: Kyoto encyclopedia of genes and genomes
SR: Salidroside
RT: Reverse transcriptase
qPCR: Real-time quantitative PCR
PMSF: Phenylmethylsulfonyl fluoride
SD: Standard deviation
ANOVA: One-way analysis of variance
